# Supplementary material for: Methodology for the Determination of a Process Safety Culture Index and Safety Culture Maturity Level in Industries
Source: Int J Environ Res Public Health. 2022 Feb 25;19(5):2668. doi: 10.3390/ijerph19052668 (PMC8909995; doi:10.3390/ijerph19052668)
Supplement: Supplementary file 1 [file ijerph-19-02668-s001.zip › ijerph-1604838-supplementary.pdf]

Supplementary material: Short version of safety culture questionnaire - selected questions

1. LEADERSHIP FOR SAFETY

1.1 Mark the sentence that describes in the best away the safety values recognized by the company.

- There are safety values up-to-dated and they influence the activities undertaken in the company
- There are some safety values and they have some influence
- Maybe there are, or not, safety values and has no influence

1.2 To what extent do you feel empowered or expected to take action to prevent injury and to keep yourself and others safe? This includes stopping work, shutting down equipment or installations, making suggestions, or taking steps to improve safety at work.

- Fully authorized
- Moderately authorized
- Not very authorized
- No authorization at all

1.3 When you take action, such as stopping work, shutting down equipment or installations, or taking steps to improve work and/or process safety, do you know that your action will be supported by your direct supervisor?

- Yes
- No

1.4 To what extent are your organization's safety policies adhered to?

- Without exception
- In general, they are respected
- Sometimes they are respected
- They are usually not followed
- Occasionally they are respected or not

1.5 How this phrase is true in your organization: "Initiatives related to modified duty and return to work include intensive efforts to rehabilitate and find meaningful temporary responsibilities for affected workers who cannot perform their regular work".

- I strongly agree
- I agree
- Neutral (partially agrees)
- Disagrees
- Definitely not true

1.6 How well do you know your organization's safety goals and safety performance indicators?

- Full knowledge
- I know, but only this applies to my department (part of the company, branch, etc.)
- I have general knowledge
- Lack of knowledge

1.7 Could you contact a board representative, board member or company owner/co-owner regarding safety?

- Yes
- No

1.8 Will direct contact with a representative of the management board, a member of the board or the owner/co-owner of the company regarding safety will be treated in the organization as a form of "denunciation"?

- No
- Yes

2. RECOGNITION AND AWARDS

2.1 To what extent can injuries be avoided throughout the organization?

- All injuries can be avoided
- Almost all of them can be avoided
- Many
- Some
- Few
- Other

2.2 To what extent can injuries be avoided in your workplace (e.g. plant, department, branch, etc.)?

- All injuries can be avoided
- Almost all of them can be avoided
- Many
- Some
- Few
- Other

2.3 Does your organization have well-established, easily accessible, stored safety values (beliefs and principles)?

- Yes
- I don't know
- No

2.4 How good the following sentence describe your organization: "In my organization, supervisors and managers have a responsibility to prevent process safety injuries and incidents in their area, and safety performance has a direct impact on their performance evaluation, promotion and wages."

- Totally agree
- True
- Doesn't really matter
- Does not affect
- Totally disagree

2.5 What is the quality of the safety rules in your organization? Quality policies are those that are up-to-date and written clearly and help people do their jobs well and safely.

- Outstanding
- Good
- Sufficient
- Weak
- Very weak
- I don't know

2.6 How is disciplinary action taken when people do not follow the safety rules of the organization? "Disciplinary action" may include a verbal warning or a more severe action such as termination.

- For all offenses

- Only in the event of serious violations
- Inconsistently
- Rarely

2.7 How would you rate the safety of physical objects (installations, devices etc.) in your work area?

- Outstanding
- Good
- Sufficient
- Weak
- Very weak. Bad

3. COMMUNICATION AND INFORMATION FLOW

3.1 Assess the effectiveness of the workplace safety structures (safety team, systems, organizational procedures, etc.)

- Outstanding
- Good
- Sufficient
- Weak
- Very weak. Bad

3.2 If you would like and could contact the executive management within company in an important safety matter, how many people would have to be informed (your direct and indirect superiors)?

- I can directly.
- Indirectly through 1 person
- Indirectly through 2 people
- Indirectly through 3 people
- More than 3 people

3.3 On average, I had to wait for my superior's response to my safety-related report or improvement (working days):

- 1 day
- 2 day
- 3 days
- 4-7 days
- >7 days

3.4 If you make a safety request, how many people must be notified to take action on the issue?

- 1 person
- 2 person
- 3 person
- 4 person
- ≥ 5 days

4. NETWORKING

4.1 In the past two years, have you been part of a safety team or task-oriented team to solve a safety problem/issue? For example, an occupational safety and health committee, a policy/procedure committee, a safe driving committee, or a task-oriented team to review the area safety regulations.

- Yes
- No

4.2 To what extent are you personally involved in organized, regularly scheduled safety audits (work surveillance) and workplace inspections?

- I am regularly involved.
- I am involved occasionally
- I am never involved

5. PERSONAL KNOWLEDGE AND SKILLS

5.1 During the last year, how active were you in safety improvement activities, such as participation in a team responsible for work safety (including process safety), participation in investigation of causes or remedial actions as a result of an incident, or assistance in the development of safety rules?

- Often
- Sometimes
- Occasionally
- Few times, maybe once in the last year or two
- Not at all, never

5.2 How many changes/improvements have you reported (regarding procedures, devices, technological processes or other improvements) with respect to the safety improvement within last year?

- 0
- 1
- 2
- 3
- ≥ 4

5.3 How many of these changes have been positively accepted within last year?

- 0
- 1
- 2
- 3
- ≥ 4

5.4 How many of these changes have been incorporated within last year?

- 0
- 1
- 2
- 3
- ≥ 4

6. CARE FOR YOURSELF

6.1 Indicate the priority you personally give to safety in your work:

- Many things are more important
- Fourth place
- Third place
- Second place
- First place. Safety comes first

7. LOSS OF ORGANIZATION IMAGE

7.1 At what point does improving safety cost more than the economic benefits it provides? (The possible economic benefits related to safety are reduced injury costs and lost working time, better morale and product quality, better production, etc.)

- There are no such restrictions
- If we achieve a very good level of security
- If we achieve a good level of security
- If we achieve an average level of security
- Always the net cost comes first

8. FATAL ACCIDENT RATE

8.1 About how many fatalities do you know in company within last year?

- 0
- 1
- 2
- 3
- ≥ 4

8.2 About how many fatalities do you know in company within last 2-3 years?

- 0
- 1
- 2
- 3
- ≥ 4

8.3 About how many fatalities do you know in company within last 4-5 years?

- 0
- 1
- 2
- 3
- ≥ 4

9. LOSS OF WORKING TIME

9.1 In how many accidents/incidents have you been involved in last 1-5 years?

- 0
- 1
- 2
- 3
- ≥ 4

9.2 In how many accidents/incidents have you been involved in last 6-10 years?

- 0
- 1
- 2
- 3
- ≥ 4

10. PERSONAL INJURIES

10.1 Do you need to use any personal protective equipment at your workplace?
- Yes
- No

10.2 Do you use any additional personal protective equipment which is not required but is delivered by employer?
- Yes
- No. Not relevant

10.3 Do you use any additional personal protective equipment which is your own property (not delivered by employer)?
- No. Not relevant
- Yes

10.4 Did you have any injuries in your workplace?
- Ye
- No

11. SAFETY TRAINING

11.1 How many formal, structured training have you received in the past two years on health and safety, and process safety?
- Many
- Enough
- Few
- Definitely not enough
- None

11.2 To what extent are the safety training, also that included in the training plan, conducted off-site?
- It is an important part/principle in the trainings being prepared
- Minor element
- There is no off-site training

11.3 Do you have enough knowledge about how to use personal protective equipment?
- More than enough
- Enough
- Too little but I can survive with that
- Definitely not enough
- None

12. CARE FOR OTHERS

12.1 To what extent in your organization safety is an integral part of the designed/ordered equipment (including devices) and facility (including installations), operational practices and work training, and not what will be added later on.
- It is the most important
- It is an integral par
- It is an essential part
- It makes up some part
- Not relevant

12.2 Indicate the priority that other employees of the organization give to safety in your opinion.
- First place
- Second place
- Third place
- Fourth place
- Not important

12.3 Do you attend safety meetings on a regular basis?
- Yes
- No

12.4 How would you rate the quality and effectiveness of safety meetings?
- Excellent
- Good
- Sufficien
- Poor
- Very weak
- None. They are not effective

12.5 To what extent are injuries, safety incidents and near-misses investigated and followed up?
- They are all investigated/analysed
- Most of them are investigated/analysed
- Many of them are investigated/analysed
- Serious incidents only are investigated
- They are rarely investigated/analysed

12.6 Have you ever reported a safety issue in your organization (description of a hazardous event, rationalization application, change of procedure, device, etc.)
- Yes
- No

12.7 After the investigated incident, in which person was injured, were the resulting recommendations applied?
- All recommendations have been applied
- Most of the recommendations have been applied
- Many of the recommendations have been applied
- Only the most important (key) recommendations have been applied
- No recommendations have been made

12.8 How do you rate the quality and effectiveness of the safety audits and inspection system? Consider the frequency, accuracy, extent of participation, the extent to which safety-related behaviours (not just physical conditions) are followed, the accuracy of follow-up, and overall effectiveness in helping to create a safer workplace
- Excellent
- Good
- Sufficient

- Poor
- Very weak. Poor

12.9 Assess the effectiveness of safety personnel (persons) in the organization (safety officer, safety advisers, safety specialists, etc.)

- Outstanding
- Good
- Sufficient
- Weak
- Very weak. Poor

13. COST OF PROACTIVE ACTIONS

13.1 How often are safety meetings held in your organization?

- Every 1-2 weeks
- Every month
- Every 2 months
- Less frequently than every 2 months
- Never

14. ORGANIZATIONAL PRIDE

14.1 Express your thoughts on how strong or long-term effects have safety improvements on such areas of the organization as quality, efficiency, costs, and profits?

- Very helpful. Significant
- Can help. Important
- Has no effect
- Makes it difficult to achieve other goals
- Disturbing to achieve other goals. Pointless
- Another

14.2 To what extent does your organization recognize safety performance and celebrate good safety performance?

- Always and very strongly emphasizes any actions that make our work safer
- Often
- Sometimes, only some very important achievements are appreciated
- Selected, but not necessarily the most essential
- Never and/or in any way

14.3 How satisfied are you with the overall level of safety in your organization?

- Very satisfied
- Moderately satisfied
- I am neither satisfied nor dissatisfied
- Partially dissatisfied
- Very dissatisfied
